# Stabilization of Human Serum Albumin by the Binding of Phycocyanobilin, a Bioactive Chromophore of Blue-Green Alga *Spirulina*: Molecular Dynamics and Experimental Study

**DOI:** 10.1371/journal.pone.0167973

**Published:** 2016-12-13

**Authors:** Milica Radibratovic, Simeon Minic, Dragana Stanic-Vucinic, Milan Nikolic, Milos Milcic, Tanja Cirkovic Velickovic

**Affiliations:** 1 Institute of Chemistry, Technology and Metallurgy - Center for Chemistry, University of Belgrade, Belgrade, Serbia; 2 Center of Excellence for Molecular Food Sciences, University of Belgrade - Faculty of Chemistry, Belgrade, Serbia; 3 Department of Biochemistry, University of Belgrade - Faculty of Chemistry, Belgrade, Serbia; 4 Department of Inorganic Chemistry, University of Belgrade - Faculty of Chemistry, Belgrade, Serbia; 5 Center for Computational Chemistry and Bioinformatics, University of Belgrade - Faculty of Chemistry, Belgrade, Serbia; 6 Ghent University Global Campus, Yeonsu-gu, Incheon, South Korea; 7 Faculty of Bioscience Engineering, Ghent University, Ghent, Belgium; University of Hyderabad, INDIA

## Abstract

Phycocyanobilin (PCB) binds with high affinity (2.2 x 10^6^ M^-1^ at 25°C) to human serum albumin (HSA) at sites located in IB and IIA subdomains. The aim of this study was to examine effects of PCB binding on protein conformation and stability. Using 300 ns molecular dynamics (MD) simulations, UV-VIS spectrophotometry, CD, FT-IR, spectrofluorimetry, thermal denaturation and susceptibility to trypsin digestion, we studied the effects of PCB binding on the stability and rigidity of HSA, as well as the conformational changes in PCB itself upon binding to the protein. MD simulation results demonstrated that HSA with PCB bound at any of the two sites showed greater rigidity and lower overall and individual domain flexibility compared to free HSA. Experimental data demonstrated an increase in the α-helical content of the protein and thermal and proteolytic stability upon ligand binding. PCB bound to HSA undergoes a conformational change to a more elongated conformation in the binding pockets of HSA. PCB binding to HSA stabilizes the structure of this flexible transport protein, making it more thermostable and resistant to proteolysis. The results from this work explain at molecular level, conformational changes and stabilization of HSA structure upon ligand binding. The resultant increased thermal and proteolytic stability of HSA may provide greater longevity to HSA in plasma.

## Introduction

**Fig 1:**
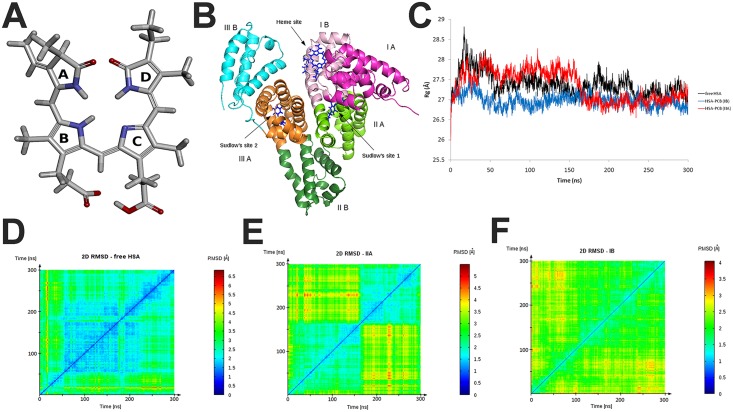
A) PCB chemical structure with labeled (A-D) pyrolle rings (oxygen atoms are in red and nitrogen in blue); B) Schematic of HSA structure with domains and ligand binding sites labeled; Radius of gyration (Rg) values (C) and 2D RMDS plots for free HSA (D), HSA-PCB(IIA) (E), and HSA-PCB(IB) (F) during 300 ns molecular dynamic simulation.

*Spirulina* (genus *Arthrospira*), filamentous blue-green microalga, has been used as food for centuries, as it is one of the richest known sources of proteins, vitamins, macro- and micro-nutrients and essential fatty acids [1]. Numerous *in vitro* and *in vivo* studies have shown the various health benefits of *Spirulina*, mainly attributed to calcium spirulan and C-phycocyanin (C-PC) [2]. Phycocyanobilin (PCB), the non-protein component of C-PC, is open-chain tetrapyrrole chromophore (Fig 1A), responsible for the intense blue color of the protein. PCB, whose structure is similar to that of biliverdin, is metabolized to phycocyanorubin by biliverdin reductase, in a manner similar to that of biliverdin conversion [3]. PCB has been shown to be a potent inhibitor of NADPH oxidase, the major source of intracellular oxidative stress [4]. PCB has strong antioxidant and anti-inflammatory effects, being able to mimic biliverdin function. Since the availability of biliverdin supplies could be inadequate for large population groups, both C-PC and PCB have been proposed as its therapeutic replacements to prevent cancer and many other diseases [4].

One of the most extensively investigated proteins, human serum albumin (HSA) is an important carrier of many physiological ligands and bioactive compounds, such as fatty acids, steroids, vitamins, metabolites and different drugs. HSA is a 66 kDa protein with three main domains (Fig 1B): domain I (residues 1–195), domain II (196–383) and domain III (384–585). Each domain of HSA is divided into two subdomains A and B containing 6 and 4 helices, respectively [5,6].

Among the seven distinct fatty acid binding sites of HSA [7], two main binding sites have been reported and first characterized by Sudlow *et al*. [8]. Sudlow’s binding site 1, known as the warfarin-azapropazone site, located in the IIA subdomain, is capable of accommodating bulky heterocyclic anion ligands. The aromatic compounds, including ibuprofen, flurbiprofen and diazepam, are found to bind in the hydrophobic pocket of Sudlow's binding site 2, located in the subdomain IIIA, known as the indole-benzodiazepine site [8]. Recent spectroscopic studies have shown the versatility of HSA molecule to accommodate both anionic and neutral molecules in Sudlow's binding site 1 [9]. Additionally, HSA has another important ligand-binding site known as heme-binding site in subdomain IB [10].

Despite numerous studies, there is controversy about the location of the primary high-affinity bilirubin-binding site on albumin. A crystallographic study placed the binding site for bilirubin photoisomer, 4*Z*,15*E*-bilirubin, within the subdomain IB [11], while numerous studies pointed to the hydrophobic pocket of the subdomain IIA as the primary high-affinity binding site for bilirubin [12]. We showed that binding sites for PCB are located in IB (heme site) and IIA (Sudlow's site I) subdomains, similar to that of its structural homolog bilirubin, and that due to its high affinity PCB competes with bilirubin for binding to HSA [13].

High flexibility consequent to ligand binding and an extraordinary capacity to accommodate a structurally diverse set of ligands at different binding sites, make HSA a non-uniform ligand-binder, capable of changing binding site conformations. Abundance of this molecule in plasma makes it a prominent factor in the pharmacokinetic behavior of a large variety of drugs, as it can cause changes in their efficiency and delivery rate [14]. Therefore, it is essential to understand how this major plasma carrier protein, capable of binding ligands at different binding sites, changes its conformation and stability upon binding a particular ligand at its specific binding site. Binding of food-derived ligands such as vitamin B12 [15], fatty acids [16], lupeol [17], and phloretin [18] have also been reported to stabilize the conformation of HSA. However, there is no comprehensive study using computational and experimental methods, which demonstrates the stabilization of HSA structure by the binding of biologically active food component.

In order to elucidate the structural changes of HSA and PCB when ligand is bound in two different (IB and IIA) HSA binding sites, the three systems: free HSA, PCB bound to heme site (HSA-PCB(IB)) and PCB bound to Sudlow's site 1 (HSA-PCB(IIA)) were examined by 300 ns molecular dynamics (MD) simulation. Using UV-VIS spectroscopy, circular dichroism (CD), Fourier Transform Infrared (FT-IR) spectroscopy, fluorescence spectroscopy and trypsin digestion assay, we examined the effects of PCB binding on HSA stability and conformation. Furthermore, following MD simulations of HSA-PCB complexes, we probed binding of various HSA ligands to their binding sites by molecular docking.

## Materials and Methods

### Materials

Essentially fatty acid free HSA (type A1887), and porcine trypsin (1800 U/mg) were purchased from Sigma-Aldrich (USA) and used without further purification. HSA concentration was determined using an extinction coefficient of 35 700 M^-1^ cm^-1^ at 280 nm. Protease inhibitors N-p-Tosyl-L-phenylalanine chloromethyl ketone (TPCK; ≥97.0%) and phenylmethanesulfonyl fluoride (PMSF; ≥99.0%) were from Sigma-Aldrich. PCB was purified from commercial Hawaiian *Spirulina pacifica* powder (Nutrex, USA) and quantified by measuring absorbance at 680 nm as previously described [13]. All measurements were done in 20 mM Tris buffer, pH 7.4 (except for trypsin digestion study, see below). Final concentrations of methanol in HSA-PCB mixtures did not exceed 1% (v/v). All other chemicals were of analytical reagent grade and Milli-Q water (Millipore, Molsheim, France) was used throughout the experiments.

### Absorbance spectroscopy measurements

UV-VIS absorption spectra were recorded using a NanoDrop 2000c spectrophotometer (Thermo Scientific, USA). The measurements of 18 μM PCB in the presence and absence of equimolar HSA were made in the range of 310–700 nm at 25°C.

### Circular dichroism measurements

CD experiments were performed on a Jasco J-815 spectropolarimeter (JASCO, Japan), under temperature controlled conditions (Peltier control system). Far-UV CD spectra of 18 μM HSA in the presence and absence of equimolar PCB were recorded in the range 180–260 nm, using a cell with a 0.1 mm path length and with an accumulation of three scans. Other relevant details are in S1 Text.

Monitoring of HSA thermal denaturation was performed in the temperature range 37–87°C, increasing the temperature at the rate of 4°C/min between 37 and 61°C and 2°C/min between 61–87°C. After 1 min of equilibration at each temperature, ellipticity was measured at 222 nm or far-UV CD spectra in the range 205–255 nm were recorded, using a cell with a 10 mm path length. For each spectrum, two scans at a scanning speed of 100 nm/min were averaged. Concentrations of HSA and PCB were 0.5 μM, with path length cells of 1 cm. Results were expressed as temperature dependence of percentage of initial ellipticity (at 37°C). Obtained plots were fitted with a sigmoidal function. The inflection point in the plot was taken as melting point of HSA [19].

### Fluorescence spectroscopy measurements

Fluorescence measurements were done on FluoroMax^®^-4 spectrofluorometer (HORIBA Scientific, Japan) under temperature controlled conditions (Peltier control system), with the width of the excitation and emission slit both adjusted to 5 nm and with cells of 1-cm path length. Temperature dependence of HSA fluorescence was studied in the range of 38–78°C, increasing the temperature at the rate of 2°C/min, with equilibration time for each temperature set to 1 min. Concentrations of HSA and PCB were 0.5 μM. Single wavelength emission at 340 nm or emission spectra in the range of 290–400 nm were recorded after excitation at 280 nm.

### FT-IR spectroscopy measurements

FT-IR data were obtained using a Nicolet 6700 FT-IR spectrometer (Thermo Scientific, USA) equipped with a Germanium attenuated total reflection (ATR) accessory, a thermoelectrically cooled deuterated triglycine sulfate (DTGS TEC) detector and a XT-KBr beam splitter. The protein secondary structure composition was determined from the shape of the amide I band, located around 1650–1660 cm^−1^. Fourier self-deconvolution and secondary derivative were applied to the range of 1700–1600 cm^−1^ to estimate the number, position, and areas of the component bands. Other relevant details are included in the S1 Text.

### Trypsin digestion of HSA

Trypsin digestion of HSA in the presence and absence of PCB was performed in 50 mM Tris buffer (pH 8.0) at 37°C. PCB (stock solution in methanol) was added to HSA solution at an equimolar concentration (3.8 μM). An equivalent volume of methanol was added in the control sample (HSA without PCB). Both samples were pre-incubated with 10 μM TPCK (final concentration) to prevent chymotrypsin activity. Digestion started with the addition of trypsin solution in 1 mM HCl (1 mg/mL), wherein the mass ratio of HSA/trypsin was 25. Aliquots of 60 μL were taken at 0.5, 2, 5, 10, 30, and 60 min after initiation of the incubation. Each aliquot was quenched with 1 mM PMSF (final concentration). SDS polyacrylamide gel electrophoresis (SDS-PAGE) of digests was performed under reducing conditions [20] and the gels were stained with Coomassie Brilliant Blue R-250. Gel images were scanned and the band intensities were quantified by densitometry using ImageJ software, considering the band intensity of HSA without trypsin (at 66 kDa) as 100%. The decay rate of HSA was fit to an exponential model with Origin software (USA), using the equation: I = a + b e^−kt^, where I and t represent relative band intensity and time, respectively, while k is rate constant of digestion. The rate constant, as well as parameters a and b, were determined by the fitting process, and after that, I was substituted with 0.5 in order to calculate the protein half-life.

### Computational details

The initial HSA receptor model was the crystal structure with PDB ID: 1BM0. The protonation state of each titratable amino acid was estimated using the H++ program by finite difference Poisson-Boltzmann (FDPB) continuum electrostatics method [21]. The AutoDock Vina program (version 1.1.2) was used for all dockings [22]. The exhaustiveness parameter was set to 100. The ligand was assigned CGenFF force field charges and non-bonded parameters, while other atom parameters were obtained from Hessian, calculated after geometric optimization on the 6-31G* level of theory by Gaussian 09 [23]. All parameters were generated using the VMD 1.9.1 program. All MD simulations were performed by applying the NAMD 2.9 program [24]. Starting structures were solvated in a periodic box of TIP3P water, followed by neutralization (by adding 150 mM NaCl) to simulate physiological conditions. A CHARMM27 force field [25] was used for protein and water. The obtained system was set to cascade 10,000 steps minimization on 310 K, followed by 250 ps equilibration in NVE ensemble with 1 fs step size. Next, the system was set to 300 ns production run in NPT dynamics using the Langevin piston pressure control at 310 K and 1.01325 bars. The Langevin damping coefficient and piston decay was set as 1 ps^−1^ and 10 fs, respectively. Periodic boundary conditions and the Particle-mesh Ewald method were implemented for a complete electrostatic calculation. The cutoff for non-bonded interactions was set to 9 Å, with switching function at 8 Å. The non-bonded list generation was stopped at 11 Å. The production phase was carried out with time step of 2 fs. The trajectory was stored every 1 ps and further analyzed with the VMD (version 1.9.2) [26] using Tcl scripts for root-mean-square deviations (RMSD), root-mean-square fluctuations (RMSF), and radius of gyration (Rg). A window-size of 20 frames with moving trajectory was used for RMSF calculations.

Molecular graphics were created using Discovery Studio Viewer 3.5 (Accelrys Software Inc. (2007) Discovery Studio Viewer, Release 3.5. Accelrys Software Inc., San Diego; http://accelrys.com/products/discovery-studio/).

## Results

### MD simulation of HSA and HSA-PCB complex

Molecular dynamics simulation is the most comprehensive computational method for predicting the time assessment of a molecular system of interacting particles; it can also supply knowledge on the fluctuations and conformational alterations of macromolecules. Extensive MD study was conducted to examine the stability of HSA and its complexes with PCB by evaluating RMSD, RMSF, and Rg. All values of the atoms in the unliganded and liganded protein in respect to the initial structures were calculated after global peptide backbone alignment up to 300-ns trajectories for all systems. In addition, 2D RMSD plots representing RMSD values between each frame of the simulation (therefore 2D) were analyzed for observing the conformational changes of HSA and its complexes with PCB during simulation. In our previous study [13], we computationally predicted two binding sites for PCB on HSA, identical to those of bilirubin (subdomains IB and IIA). The MD simulation study enabled us to refine these PCB-binding sites and to better define interactions at both binding sites (S1 Fig, S1 Table and S2 Text).

RMSD values of the Cα-atoms of HSA and HSA-PCB complexes indicated that all systems reached equilibrium, oscillated around an average value, and remained stable until the end of the simulation (Figure A in S2 Fig). According to the 2D RMSD plot (where the squares along the diagonal of the plot indicate intervals of snapshots having structural similarities), free HSA passed through three different conformations (Fig 1D). From the start of 300 ns MD simulation, HSA resided in a single conformation for about 50 ns, and then transitioned into a conformation distinct from the first one. It held this (or similar) structure until 230 ns, and then transitioned into its final conformation. The light blue crosses (localized peaks in RMSD value) indicate that the final conformation has structural similarities to the intermediate state, but not to the starting conformation. HSA adopted only two clearly distinct conformations when PCB was bound at the IIA site (Fig 1E). It transited from one conformation to another at 170 ns, and there were low structural similarities between these conformations. In contrast, HSA having PCB bound at site IB retained its starting conformation until the end of simulation (Fig 1F). Thus, 2D RMSD analysis indicates HSA rigidification upon PCB binding, with a more prominent effect for binding of PCB at site IB.

The Rg value is a measure of the compactness of the system during simulation. In contrast to free HSA, which showed a fluctuation in Rg during the entire simulation (Fig 1C), HSA with PCB bound to site IIA clearly reached equilibrium at about 170 ns, at which it adopted its final conformation, as indicated by the 2D RMSD analysis (Fig 1F). The lower plot of HSA-PCB(IB) during the entire simulation period implies that this form may have a noticeably more compact conformation, in comparison to free HSA and HSA-PCB(IIA). Therefore, in parallel with RMSD, Rg analysis suggests that liganded HSA is more stable than its free form, and that PCB binding at site IB stabilizes HSA from the start, while binding at site IIA accelerates it to reach equilibrium.

**Fig 2:**
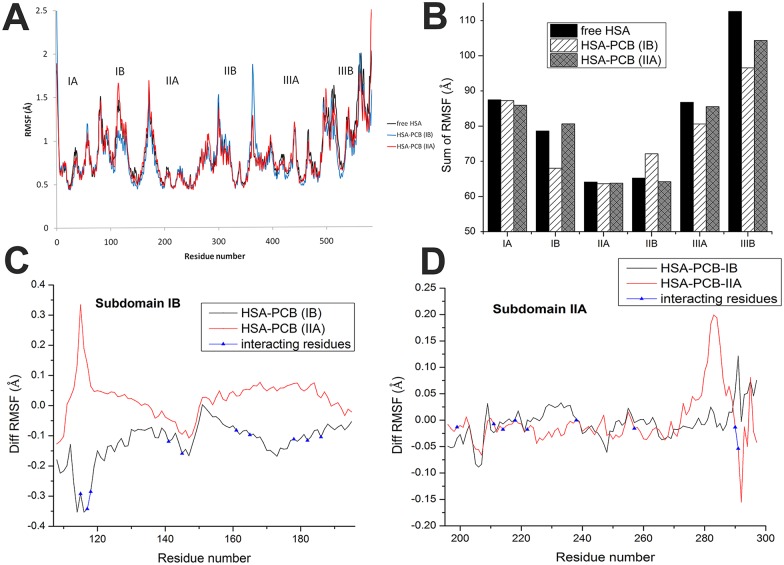
Calculated RMSF values (Å) from molecular dynamics simulation of free HSA, HSA-PCB(IB), and HSA-PCB(IIA). RMSF values along whole protein sequence (A), sum of RMSF values of each HSA domain (B), and difference of RMSF values between PCB-HSA complexes and free HSA, with marked amino acid (aa) residues involved in PCB binding for site IB (C), and for site IIA (D).

The local protein mobility was analyzed by calculating the time-averaged RMSF values of pure HSA and both HSA-PCB complexes. Generally, the atomic fluctuations were found to be very similar to those of pure HSA and the HSA-PCB complexes (Fig 2A). However, local changes upon binding of the ligand occurred not only in the binding sites but also in the distant regions of the protein, which may suggest an overall conformational change of the protein. Binding of PCB to the IB-binding site caused a decrease in RMSF of subdomain IB (Fig 2C). Residues (115–186 aa region) involved in PCB binding at site IB (S2 Text and S1 Table) synergistically rigidified the whole IB region. Although binding of PCB to site IIA did not notably rigidify the IIA subdomain, there was an increase in flexibility in the region (270–285 aa) that is not involved in binding (Fig 2D). The most pronounced rigidification was observed in subdomain IIIB in both examined systems with bound PCB, with a greater effect when PCB was bound to subdomain IB (Figure D in S3 Fig). Binding of PCB to the site located in subdomain IB caused a pronounced increase in the RMSF of residues in subdomain IIB (350–375 aa) (Figure B in S3 Fig), while binding of PCB to the site IIA caused a slight increase in flexibility in subdomain IB (100–120 aa) (Fig 2C).

As PCB binding increased the rigidity of some HSA regions and increased the flexibility of the others, to get a quantitative insight into the flexibility of individual domains in all three systems, we calculated the sum of RMSF values of all amino acid residues for every domain (Fig 2B). During the MD simulation of free HSA, subdomain IIIB showed the highest flexibility, twice as high as that of subdomains IIA and IIB. PCB binding at site IB induced an increase of 13% and 14% in the rigidity of subdomains IB and IIIB, respectively, and an increase of 10% in the flexibility of subdomain IIB. PCB binding at site IIA induced a prominent increase of 7% in the rigidity of subdomain IIIB. It is interesting to note that increased rigidity of subdomains IIIB and IB, when PCB bound at IB site, was also the consequence of an interaction between a loop in subdomain IB and an α-helix in subdomain IIIB (Figure A in S4 Fig). Positioning of PCB at site IB allowed the formation of alternating hydrogen bonding of Arg114 (IB) with Glu520 and Ala511 (IIIB), resulting in the stabilization of both subdomains IB and IIIB (Figure B in S4 Fig). In free HSA, there was almost no interaction between the two subdomains (Figure D in S4 Fig); when PCB was bound at site IIA, there was a transitory (about 100 ns) interaction (Figure C in S4 Fig), resulting in lower stabilization, as compared to that seen in HSA-PCB(IB).

**Fig 3:**
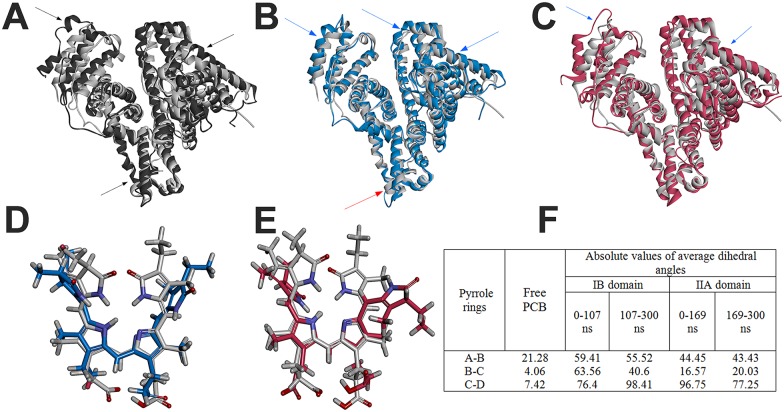
**Conformational changes of free HSA (A), HSA-PCB(IB) (B), and HSA-PCB(IIA) (C) during molecular dynamics simulation**. The starting structures (0 ns) are represented in grey and the structures from the last snapshot (300 ns) in black (free HSA), blue (HSA-PCB(IB)) and red (HSA-PCB(IIA)). The red arrows direct to regions of PCB-bound HSA with increased flexibility, and blue one to decreased flexibility compared to flexibility of free HSA (black arrows); **Conformational changes of PCB bound to site IB (D) and site IIA (E) during molecular dynamics simulation**. The starting structures (0 ns) are represented in grey and the structures from the last snapshot (300 ns) in blue (PCB-IB) and red (PCB-IIA); **(F) Absolute values of dihedral angles of starting PCB structure and of average dihedral angles calculated before and after PCB conformational change (at 107 ns for PCB-IB and at 169 ns for PCB-IIA)**.

Taking into account RMSF values for all HSA residues, PCB binding to site IB and IIA caused an increase of 5.4% and 2.1%, respectively, in the overall rigidity of HSA, suggesting that PCB binding at any two sites stabilizes the protein structure. These changes in RMSF are also reflected in the movement of subdomains in HSA, as shown in Fig 3A, 3B and 3C.

Overall, the MD simulation data suggest a conformational change in HSA upon binding of PCB, which can influence the binding sites for other ligands (especially heme-binding site may be influenced upon PCB binding to Sudlow's site I). According to the MD simulation data, the binding of PCB also causes greater compactness of the protein and reduces its conformational mobility, suggesting that HSA, when complexed with PCB, is more stable than the free protein.

### Phycocyanobilin bound to HSA undergoes a conformational change to a more stretched conformation

To get insight into conformation of ligand during the MD simulation, RMSD, RMSF and dihedral angles between pyrolle rings of PCB were calculated. The RMSD of the ligand reached equilibrium after 110 ns and 170 ns when bound to IB and IIA sites, respectively (Figure B in S2 Fig). In comparison with values of the protein backbone, ranging from 2.5–4.5 Å (Figure A in S2 Fig), RMSD of bound ligand ranged from 1.7–3.5 Å, indicating that protein structure shows higher fluctuations then ligand, in accordance to the high affinity of the ligand, and suggests that the docked complex structure in both cases is stable throughout the MD simulation. When PCB is bound at site IIA, dihedral angle between rings A and B is stable during simulation (Figure A in S5 Fig), as well as angle between rings B and C (Figure B in S5 Fig). However, dihedral angle between rings C and D (Figure C in S5 Fig) dramatically change at 169 ns, at the same time at which HSA change its conformation (Fig 1C and 1E), suggesting simultaneous conformational change of protein and ligand. On the contrary, when PCB is bound at site IB all dihedral angles become stabilized after 107 ns (S5 Fig). In both cases angles between rings B and C are the most stable (Figure B in S5 Fig). RMSFs of all heavy atoms of PCB (Figure A in S6 Fig) are similar when PCB is bound to IB or IIA site (Figure B in S6 Fig), with atoms of the pyrrole rings showing the lowest flexibility. Atoms of internal rings (B and C) are more rigid then external ones, with central bridge atoms showing far the lowest fluctuation, in accordance to observed the highest stability of dihedral angles between rings B and C. These results suggest that PCB is stable protein-bound during simulation time and that it undergoes a conformational change in the binding site.

In order to determine conformations of PCB bound to HSA, dihedral angles between pyrrole rings of PCB were calculated using data obtained by MD study. Average absolute values of dihedral angles during 300 ns MD simulation, before and after PCB conformational change (at 107 ns for HSA-PCB(IB) and at 169 ns for HSA-PCB(IIA)) are shown in Fig 3F. Dihedral angles of 0°-90° can been ascribed to *syn* (*S*) conformers, while angle of 90°-180° represents *anti* (*A*) conformers. Taking into account that free PCB has *SSS*, cyclic, helical conformation [27] values at Fig 3F indicate that PCB bound to HSA has more stretched conformation in comparison to its free form. Conformer at subdomain IIA resides in *SSA* conformation until its conformational change at 169 ns, while conformer at subdomain IB adopts elongated SSA conformation after its conformational change at 107 ns, therefore being more elongated at the end of simulation. Overlaid structures of bound PCB before and after MD simulation are shown at Fig 3D and 3E.

**Fig 4:**
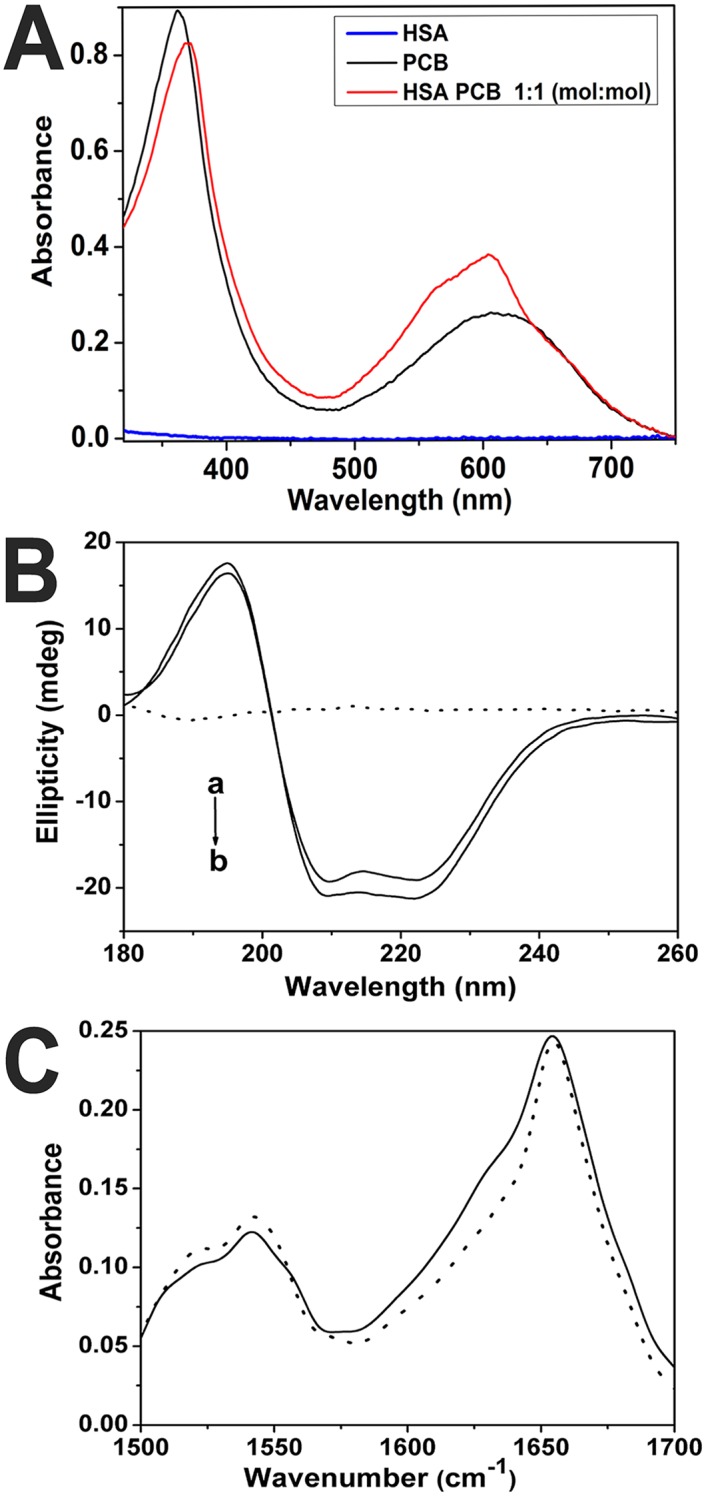
**(A) UV-VIS spectra of free HSA, free PCB, and HSA-PCB complex (molar ratio 1:1)**; **(B) Far-UV circular dichroism spectra of HSA in the absence (a) and presence (b) of PCB**. Dot line curve shows spectrum of PCB; **(C) The FT-IR spectra of free HSA (solid line), and the FT-IR difference spectra of HSA-PCB complex (molar ratio 1:1) (dot line)**. All spectra were recorded in 20 mM Tris buffer (pH 7.4) with samples of 18 μM HSA and 18 μM PCB.

The UV-VIS spectrum of pure PCB shows two characteristic absorption maxima at 362 and 610 nm (Fig 4A). Addition of HSA results in shift of absorption maximum from 362 nm to longer wavelengths (up to 368 nm) with decreasing absorbance and an increase in the intensity of absorbance in VIS region, followed with blue shift to 605 nm. These observations confirm that PCB conformation changes after addition of HSA.

### HSA conformation changes by interaction with PCB

The CD spectra of HSA displays two negative bands in the UV region at 208 nm and 222 nm, characteristic of the α-helical structure of protein. As shown in Fig 4B, PCB alone does not show optical activity in this region. The addition of PCB to HSA leads to an increase in negative ellipticity of minima without any significant shift of the peaks or overall protein spectrum shape change. The α-helix content of HSA increased from 49.3±1.7% to 54.5±2.4% upon PCB binding at a molar ratio PCB/HSA of 1:1, implying that PCB binding results in conformational change that stabilizes HSA structure by increasing its α-helix content.

Additional evidence regarding the effect of PCB binding on HSA structure came from FT-IR study (Fig 4C, S3 Text and S7 Fig). Free HSA contains α-helices (50.4±2.1%), β-sheets (29.8±1.3%), and β-turns (11.0±0.5%). These results are consistent with the literature data [28]. Addition of PCB to HSA, increased the α-helix content to 55.2±1.8%, confirming the results obtained by CD. The total β-sheet and β-turn content was virtually unchanged; however, the random coil content was reduced from 8.8±1.3% to 4.8±0.7% (S2 Table), suggesting that upon PCB binding HSA stabilization is reflected by increase in α-helix content on the expense of random coil.

### Increase in thermal stability of HSA by PCB binding

**Fig 5:**
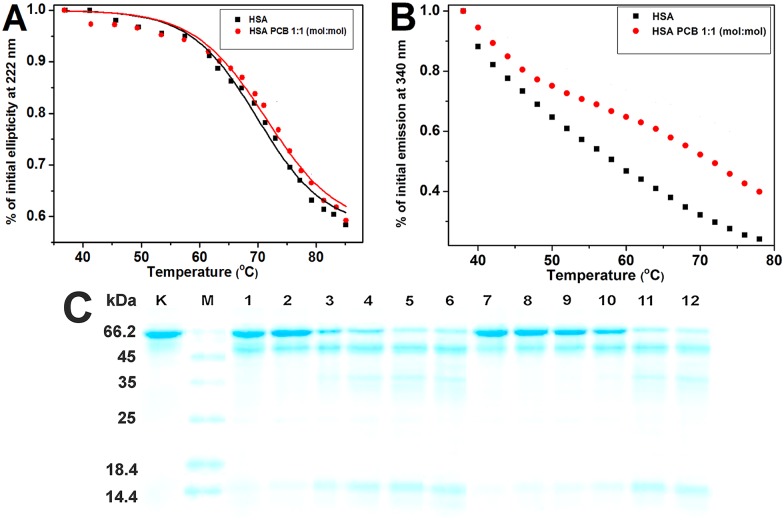
(A) Temperature dependence of 0.5 μM HSA ellipticity at 222 nm in the presence and absence of 0.5 μM PCB; (B) Temperature dependence of 1 μM HSA fluorescence at 340 nm in the presence and absence of 1 μM PCB (λ_EX_ = 280 nm); (C) SDS-PAGE profile after trypsin digestion of 3.8 μM HSA in the presence and absence of 3.8 μM PCB. Lane K: HSA without trypsin; lane M: MW markers; lanes 1–6 correspond to digestion times 0.5, 2, 5, 10, 30, and 60 min without PCB, respectively; lanes 7–12 correspond to digestion times 0.5, 2, 5, 10, 30 and 60 min with PCB, respectively.

In order to investigate the effect of PCB binding on HSA thermal stability, we monitored its thermal denaturation by decrease of ellipticity at 222 nm because of α-helical content loss (Fig 5A). The melting curve for HSA-PCB system was similar to that of free HSA; however, it was observed that PCB has limited inhibitory effect on HSA thermal denaturation. Tm values obtained by sigmoidal fit of melting curves indicate that compared to free HSA (Tm = 70°C) HSA-PCB complex has greater thermal stability (Tm = 71.4°C). In addition, far-UV CD spectra recorded at each temperature also show increased HSA thermal stability by PCB binding (Figures A and B in S8 Fig). Thermal denaturation was also reflected by decrease in intrinsic fluorescence of Trp214 located in IIA subdomain [6] (Fig 5B). However, unlike CD, intensive decrease of fluorescence starts at lower temperatures (40°C). Therefore, it appears that fluorescence loss at lower temperatures may not be a consequence of protein conformational changes because thermal quenching also occurs here [29]. However, there are differences in melting curves of free HSA and HSA-PCB complex, especially at temperatures above 60°C. The presence of PCB inhibits decrease in HSA fluorescence at higher temperatures, indicating that PCB stabilizes HSA structure. Furthermore, the change in the fluorescence emission spectra (λ ex = 340 nm) recorded at each temperature shows increase in thermal stability of HSA by PCB binding (Figures C and D in S8 Fig).

### Resistance of HSA-PCB complex to trypsin digestion

Flexibility of protein domains influences protein digestion because flexible loops of proteins can better accommodate catalytic sites of proteases. As the HSA-PCB complex is more rigid than the free HSA, it might be expected that the ligand binding will influence the proteolytic degradation of the protein.

HSA digestibility in the presence and absence of PCB was analyzed by SDS-PAGE after trypsin digestion. The results demonstrate that trypsin in 10 min digested majority of free HSA (Mw 66 kDa), producing relatively large fragments. On the other hand, HSA-PCB complex is digested slower than HSA, and the large digestion resistant peptides of HSA (Mw of 35 and 14 kDa) appear later during the digestion reaction (Fig 5C). By fitting the intensity of the intact HSA band obtained by densitometry into an exponential decay curve (S9 Fig), we estimated half-life of free HSA to be 4.9 min, while PCB prolonged half-life of HSA to 13.9 min.

### Complexation of PCB with HSA affects the binding of other physiological ligands

MD simulation study of conformational changes occurring upon binding of PCB to any of its two possible binding sites on HSA allowed us to theoretically investigate effects of multiple ligands binding to HSA or HSA in a complex with PCB. We inspected several physiologically relevant ligands: bilirubin, warfarin, indomethacin, fusidic acid, thyroxine, diazepam, ibuprofen, diclofenac, hemin, azapropazone, myristic acid, and PCB. The approach involved docking of the ligands to the initial protein conformation and observation of the conformation that protein adopts after MD simulation (as shown above, Fig 3A, 3B and 3C).

During MD simulation HSA changed its conformation, and therefore, binding scores of almost all ligands after simulation were lower than they were in the initial crystal structure (Table 1). After MD simulation of HSA with PCB bound at site IIA, binding scores of ligands to sites IA, IB, IIIA, and IIIB were mostly reduced; however, after MD simulation of HSA with PCB bound at site IB, binding scores were either reduced or increased. It is interesting that binding scores of ligands bound to site IIB increased because of the presence of PCB at site IB or IIA. It can be concluded that conformational change induced by PCB binding to site IIA in general negatively influences the binding of different ligands to the sites IA, IB, IIIA, and IIIB. On the other hand, the conformation that HSA adopts when PCB binds to any of its two high-affinity sites is more favorable for binding of ligands that specifically bind to site IIB. Our data imply that HSA structure stabilization by PCB binding and the accompanying conformational change might influence the binding of other ligands (including PCB) to other binding sites.

**Table 1:** Binding scores (kcal mol^-1^) of different ligands docked to free and phycocyanobilin (PCB)-bound human serum albumin (HSA; at site IB or IIA), before and after 300 ns of molecular dynamics (MD) simulation.

Ligand	Binding site	Free HAS before MD	Free HAS After MD	HSA-PCB(IB) after MD	HSA-PCB(IIA) after MD	Diff free HSA after MD—HSA-PCB(IB) after MD	Diff free HSA after MD—HSA-PCB(IIA) after MD
**Warfarin**	IIA	-8.8	-7.5	-7.0	------	-0.5	------
**Indomethacin**	IB	-8.4	-7.4	------	-6.7	------	-0.7
	IIA	-8.2	-6.9	-6.9	------	0.0	------
**Bilirubin**	IB	-9.8	-7.5	------	-8.3	------	0.8
	IIA	-8.3	-7.6	-6.1	------	-1.5	------
**Fusidic acid**	IB	-8.8	-7.4	-------	-6.8	------	-0.6
	IIIB	-6.5	-5.3	-4.7	-5.3	-0.6	0.0
**Thyroxine**	IIA	-6.4	-6.0	-5.7	------	-0.3	------
	IIIA	n/a	-6.4	-5.3	-4.4	-1.1	-2.0
	IIIB	-5.3	-5.4	-3.6	-5.0	-1.8	-0.4
**Hemin**	IB	-8.2	-7.8	------	-6.6	------	-1.2
**Diazepam**	IIIA	n/a	-6.9	-7.2	-6.3	0.3	-0.6
**Ibuprofen**	IIB	-6.8	-6.2	-6.8	-6.8	0.6	0.6
	IIIA	-5.9	-6.1	-6.5	-6.4	0.4	0.3
**Azapropazone**	IB	-8.1	-7.3	------	-6.8	------	-0.5
	IIA	-8.1	-7.2	-6.3	------	-0.9	------
**Diclofenac**	IB	-7.3	-7.0	------	-6.1	------	-0.9
	IIA	-8.1	-6.2	-6.4	------	0.2	------
	IIB	-7.2	-6.6	-7.1	-6.9	0.5	0.3
**Myristic acid**	IIA	-5.9	-5.3	-5.3	------	0.0	------
	IB	-6.0	-5.6	------	-4.4	------	-1.2
	IA	-4.4	-4.2	-4.3	-4.6	0.1	0.4
	IIB	-5.6	-4.9	-5.2	-5.8	0.3	0.9
	IIIA	-6.0	-5.4	-5.6	-5.1	0.2	-0.3
	IIIB	-3.8	-4.4	-4.8	-4.5	0.4	0.1
**PCB**	IB	-10.2	-8.9	------	-8.7	------	-0.2
	IIA	-10.0	-8.5	-8.1	------	-0.4	------

## Discussion

We have shown that an algae-derived tetrapyrrole pigment, when bound to the major plasma carrier protein, stabilizes its structure and provides protection from proteolytic degradation. Our results also indicate that for proteins binding multiple ligands, conformational changes upon binding of tetrapyrrole ligands may have various effects on binding of other physiologically relevant ligands/drugs of HSA.

In aqueous solutions, PCB adopts a monoanionic helicoidal conformation with the first carboxylic group ionized [13]. Rotation about exocyclic single bonds of the methine bridges, and protonation state of propionic acid residues in PCB, produces various conformers with spectroscopically different characteristics [30], a property we exploited for analyzing conformation of the ligand bound to HSA. PCB possesses high conformational flexibility, but the cyclic helical *SSS* conformation is the most stable [27]. Binding of PCB to HSA induced changes in the absorption spectrum of PCB, with a blue shift and increase in intensity of the VIS peak and a red shift and decrease in intensity of the UV peak. This indicates that PCB bound to HSA has a different, slightly more stretched conformation in comparison to its free form, in agreement with calculated transitions for protonated, more extended PCB conformers [31]. Red shift and decrease in intensity of the bilirubin peak at 440 nm was also observed upon its binding to HSA [32]. Indeed, dihedral angles between pyrrole rings in PCB, calculated from the MD study, confirmed that PCB bound to HSA adopts a more stretched conformation at both the binding sites.

During the 300 ns of MD simulation, free HSA underwent three different conformations (initial, transitional, and final). Upon PCB binding to site IB, HSA underwent only slight conformational change, indicating that the PCB binding site IB is mainly preformed and PCB rigidifies it upon binding. In contrast, conformational adjustments of the HSA molecule by PCB binding to site IIA was more permissible; at about 170 ns, conformational adjustment was complete and the HSA structure was rigidified from that moment, although less than that in the aforementioned case. The lower plot for Rg values of HSA-PCB(IB) during the entire simulation period, and switch of Rg of HSA-PCB(IIA) to lower values after 170 ns implies that the HSA-PCB complex has more compact conformation in comparison to free HSA. Analysis of RMSF for individual subdomains demonstrated that PCB binding at site IB induces noticeable increase in the rigidity of the subdomains IB and IIIB and increases flexibility in subdomain IIB, whereas PCB binding at site IIA induces prominent increase in the rigidity of subdomain IIIB. Residues involved in PCB binding at site IB highly contributed to the rigidification of subdomain IB, whereas residues involved in PCB binding at site IIA did not allow increase in flexibility. Overall, PCB binding to site IB and IIA increased the rigidity of the whole HSA structure for cca 5% and 2% respectively, supporting other results and demonstrating that PCB binding at any of the two sites stabilizes HSA structure.

CD and FT-IR measurements showed that binding of PCB caused a slight conformational change of the protein, with an increase in its α-helical content. It has previously been reported that binding of certain ligands such as vitamin B12 [15] and virstatin [33] may cause conformational change in HSA with an accompanying increase in its α-helical content and protein structural stabilization. This phenomenon is quite striking for protein–ligand systems with high affinities, e.g., binding of biotin to streptavidin causes the disappearance of the band arising from unordered structure in FT-IR spectra [34].

CD and fluorescence melting curves of HSA in the presence and absence of PCB confirmed that PCB binding leads to an increased protein thermal stability. Increase in HSA Tm was also found upon binding of several other ligands to site IIA, such as warfarin [35], virstatin [33], and hippuric acid [36]. Previous studies have found that increase in thermal stability induced by ligand binding correlates with decrease in protein flexibility, i.e., increased protein packing induces greater stability [37]. Therefore, binding of PCB to HSA increases rigidity, and consequently, the thermal stability of HSA.

Trypsin digestion study showed that the HSA–PCB complex is more resistant to proteolysis in comparison to free HSA. This is in accordance with previous findings that the binding of some ligands to HSA, such as anionic azo-dyes [38] and bilirubin [39], a structural analog of PCB, decreases the susceptibility of HSA to hydrolytic attack by trypsin. Increased compactness and reduced flexibility of HSA induced by PCB binding protected the protein from proteolytic degradation, as susceptibility to proteolysis is determined by exposure of peptide bonds, as well as by mobility of protein segments containing these bonds. An important consequence of the observed phenomenon may be that PCB, when bound to the major carrier protein, may prolong the protein’s half-life in plasma by making it less susceptible to proteases.

Our results also indicate that for proteins binding multiple ligands, conformational changes upon binding of the tetrapyrrole ligand may have various effects on binding of other physiologically relevant ligands of HSA. There is no strict ligand specificity for the HSA-binding sites, and HSA, as an indiscriminate ligand binder, binds a variety of ligands without extremely high affinities. Each site in HSA is able to bind ligands of variable shapes and sizes owing to a flexible protein structure and ability of three HSA domains for substantial relative movement. Our study implies that PCB binding influences the binding of ligands to the other sites, mostly by reducing their binding. This can be explained by the fact that bound PCB reduces HSA flexibility, and thus limits HSA from adopting dynamic conformations necessary for other ligand fittings.

HSA acts as a carrier for drugs, peptides, and small proteins (e.g., cytokines) allowing them to circulate for as long a time as the half-life of HSA itself. Therefore, the actual concentration of drugs, peptides, and small proteins in any compartment depends on their ability to bind HSA and/or other plasmatic carrier proteins [14]. Our results directly imply that binding of PCB to HSA will not only influence the protein’s ability to bind ligands, but may also effect higher stability and longevity of the protein in plasma.

## Supporting Information

S1 Fig Main interactions in HSA-PCB at binding site IIA (A) and IB (B) after 300 ns of MD simulation. (PDF)

S2 Fig RMSD values (Å) of free and PCB-ligated HSA (A) and HSA-bound PCB (B) during 300 ns of molecular dynamic simulation. (PDF)

S3 Fig Difference in RMSF values (Å) between PCB-HSA complexes and free HSA, for subdomains IA (A), IIB (B), IIIA (C), and IIIB (D). (PDF)

S4 Fig (A) Interaction between Arg114 of the loop in subdomain IB and Glu520 of the α-helix in subdomain IIIB (up), and interaction between Arg114 of the loop in subdomain IB and Ala511 of the loop in subdomain IIIB (down); Hydrogen bond distance at HSA-PCB(IB) (B), HSA-PCB(IIA) (C), and free HSA (D). (PDF)

S5 Fig Dihedral angles of PCB bound to HSA at site IB (black) and IIA (red) during 300 ns of molecular dynamic simulation. Dihedral angles (deg) between rings A and B (**A**), rings B and C (**B**), and rings C and D (**C**).
(PDF)

S6 Fig (A) Structure of PCB with numbering of atoms indicated; (B) RMSF values (Å) of heavy and polar hydrogen atoms of PCB bound to HSA (at binding site IB or IIA) during 300 ns MD simulation. (PDF)

S7 Fig FT-IR spectra of free HSA and PCB-bound HSA. (**A**) The curve-fit amide I (1700–1600 cm^-1^) region with secondary structure determination of the free HSA, and (**B**) the curve-fit amide I (1700–1600 cm^-1^) region with secondary structure determination of HSA-PCB complex (18 μM both).
(PDF)

S8 Fig **Temperature dependence of 0.5 μM HSA far-UV CD spectra in the presence (A) and absence (B) of 0.5 μM PCB**. *Note*: *More pronounced effect of PCB-induced thermal stabilization of HSA*, *obtained on the basis of an analysis of far-UV CD spectral data*, *compared to ellipticity at 222 nm at different temperatures is due to differences in time the sample spent at each temperature*. *Ellipticity at 222 nm was recorded immediately after 1 min of mixture equilibration; CD spectral data were obtained after a total 2 min*: *1 min of equilibration*, *and 1 min of spectra recording*. **Temperature dependence of 1 μM HSA fluorescence emission spectra in the presence (C) and absence (D) of 1 μM PCB (λ ex = 280 nm)**. *Note*: *The blue shift of emission maximum in HSA-PCB sample is the result of PCB binding to HSA*, *as described in our previous study* [13].
(PDF)

S9 Fig Quantification of HSA digestion by trypsin in the presence and absence of PCB. Band intensities at 66 kDa were quantified by densitometry after SDS-PAGE.
(PDF)

S1 Table Residues involved in interactions between HSA and PCB at binding sites IIA and IB. (PDF)

S2 Table Contents of different secondary structures of HSA in the presence and absence of phycocyanobilin, obtained using FT-IR spectroscopy (n = 3). (PDF)

S1 Text Experimental details. (PDF)

S2 Text Binding sites for PCB on HSА. (PDF)

S3 Text PCB induces changes in FT-IR spectrum of HSA. (PDF)
