# Supplementary figures and images for: Stabilization of Human Serum Albumin by the Binding of Phycocyanobilin, a Bioactive Chromophore of Blue-Green Alga Spirulina: Molecular Dynamics and Experimental Study
Source: PLoS One. 2016 Dec 13;11(12):e0167973. doi: 10.1371/journal.pone.0167973 (PMC5154526; doi:10.1371/journal.pone.0167973)

**A**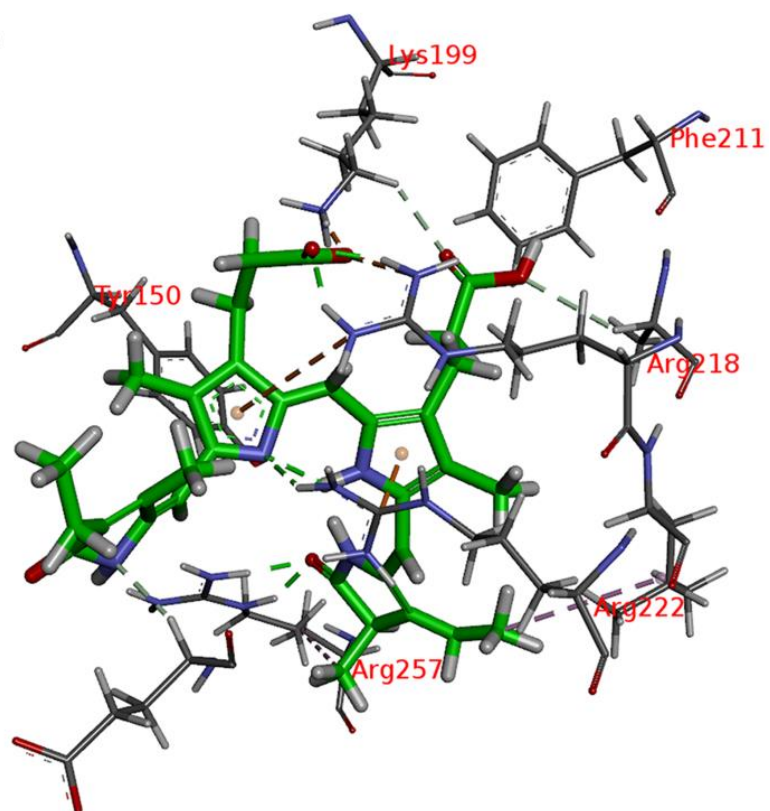**B**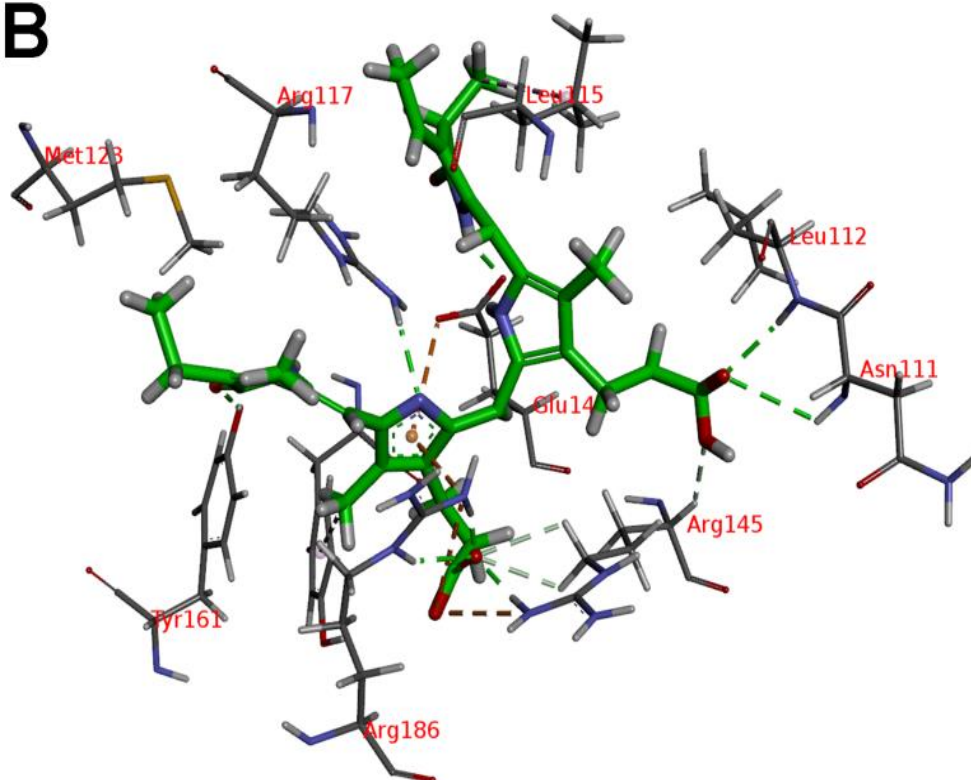

Supplement: S1 Fig — (PDF) [file pone.0167973.s001.pdf]

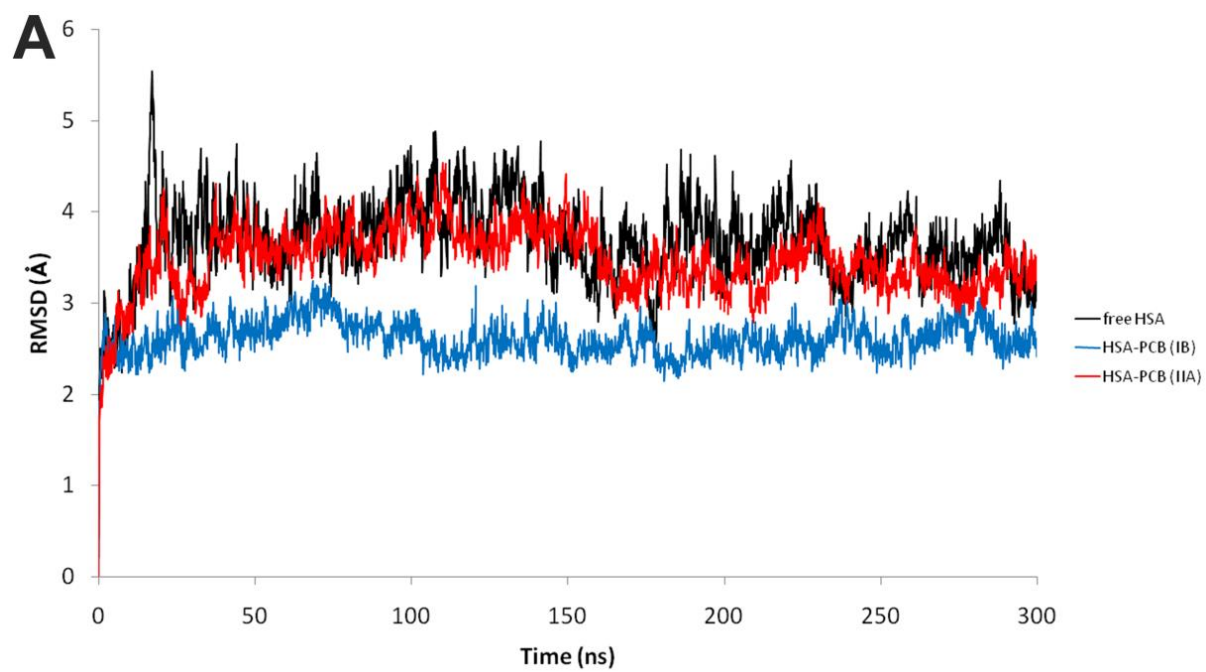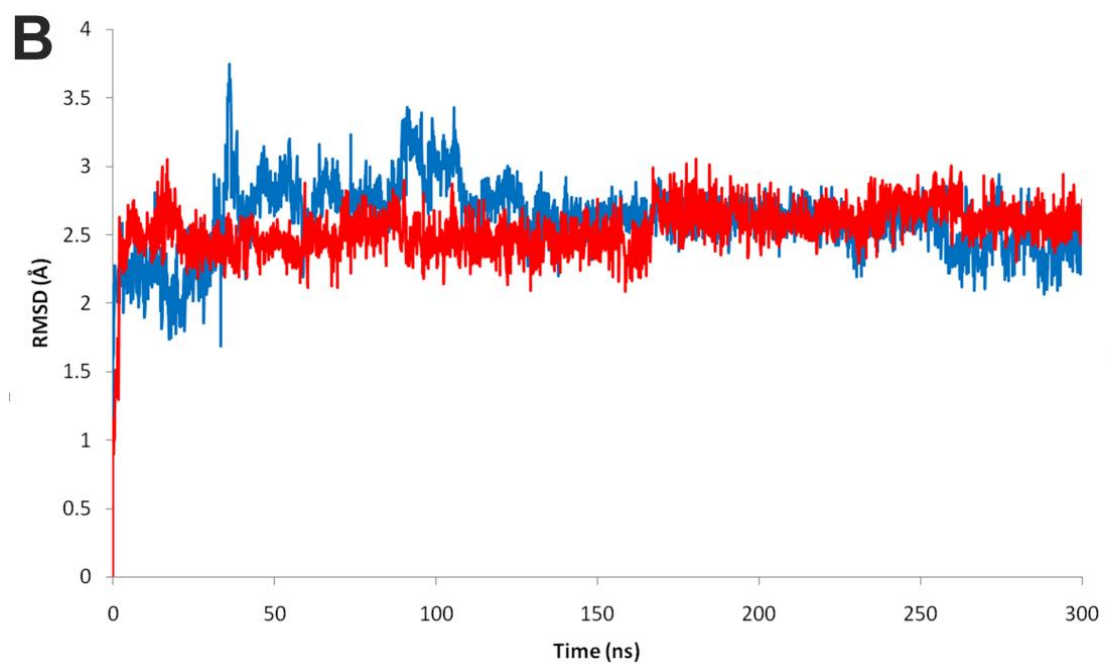

Supplement: S2 Fig — (PDF) [file pone.0167973.s002.pdf]

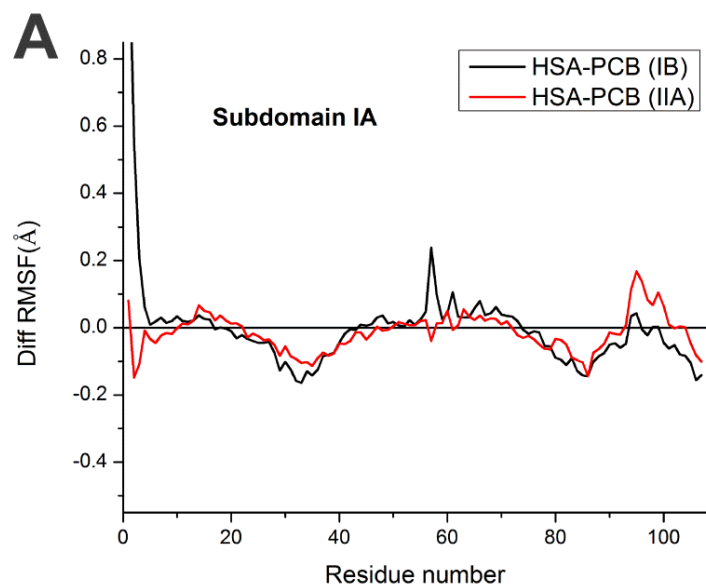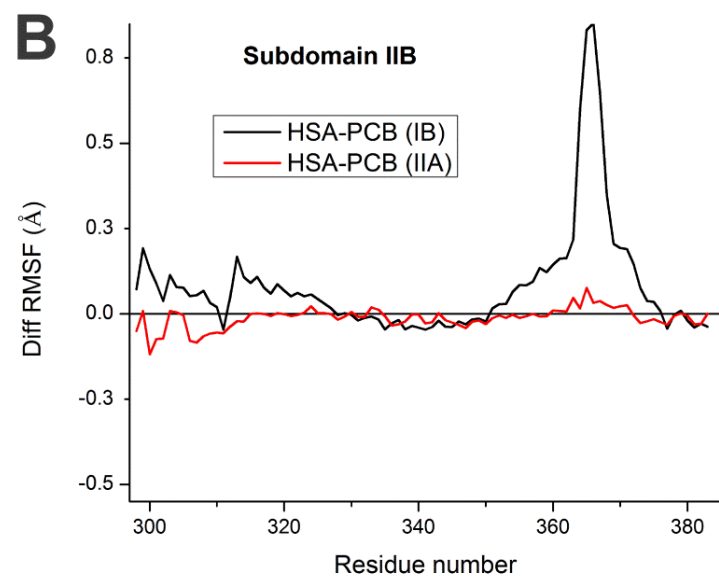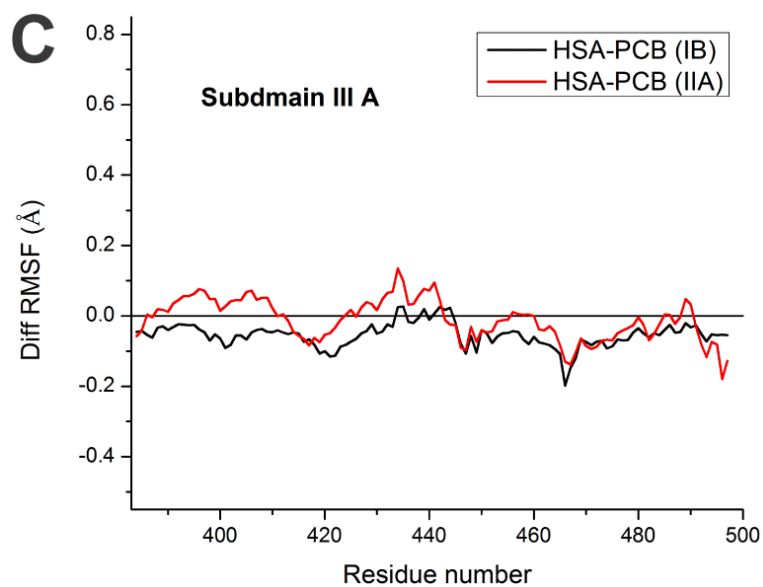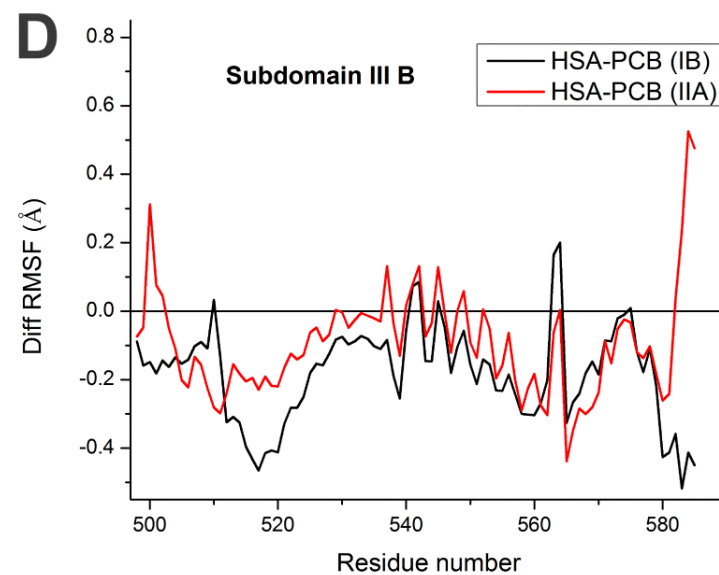

Supplement: S3 Fig — (PDF) [file pone.0167973.s003.pdf]

A

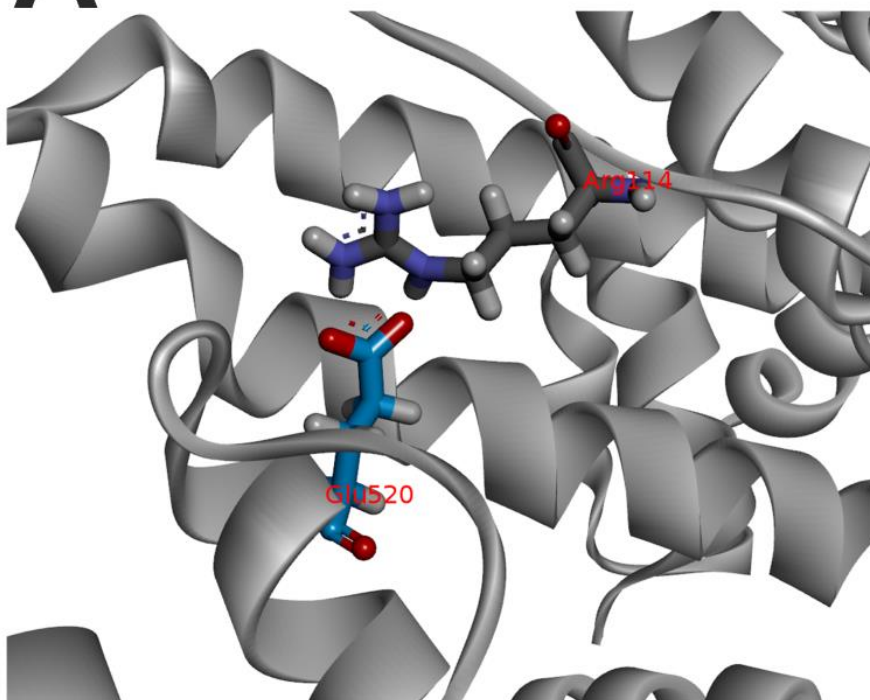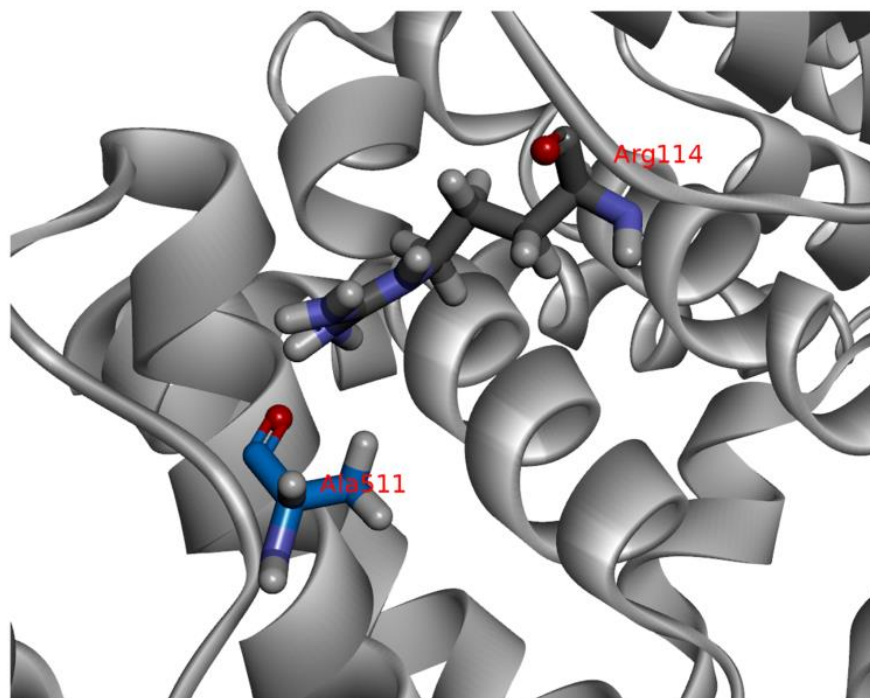

**B**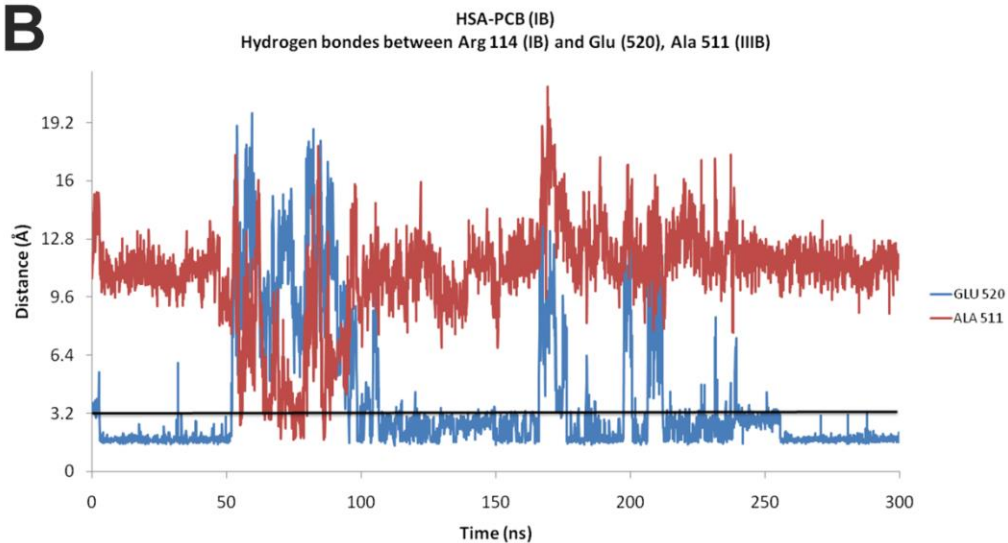**C**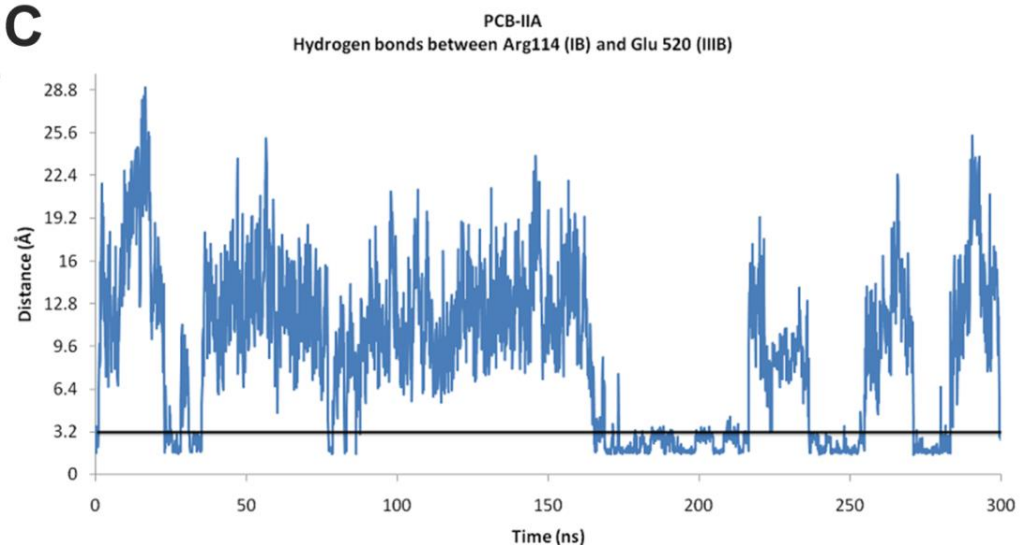**D**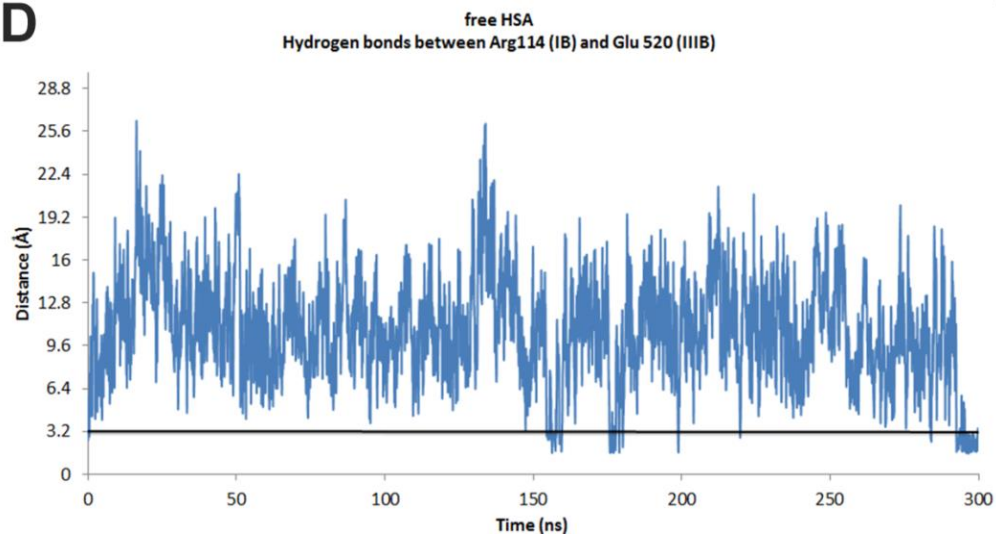

Supplement: S4 Fig — (PDF) [file pone.0167973.s004.pdf]

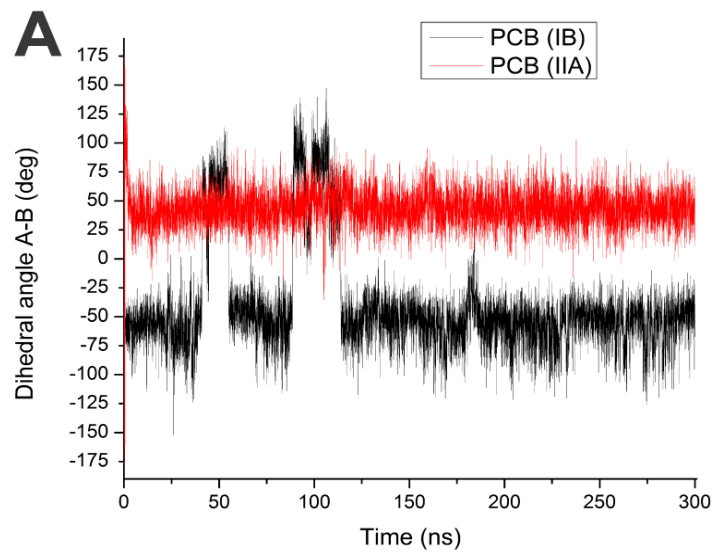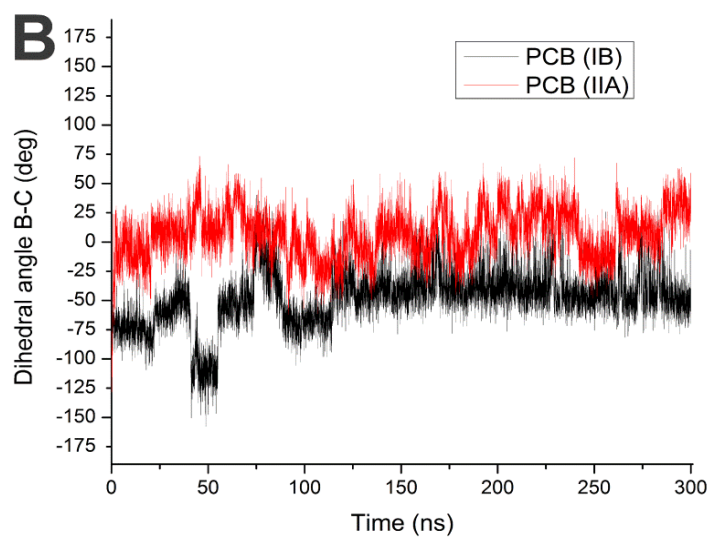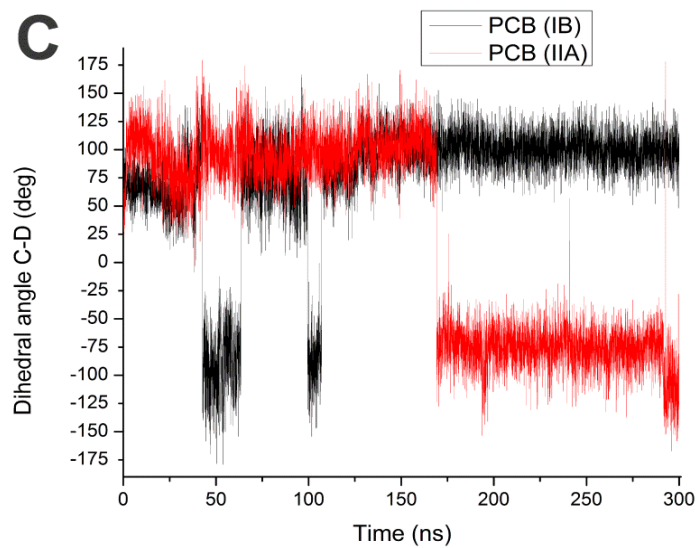

Supplement: S5 Fig — Dihedral angles (deg) between rings A and B (A), rings B and C (B), and rings C and D (C). (PDF) [file pone.0167973.s005.pdf]

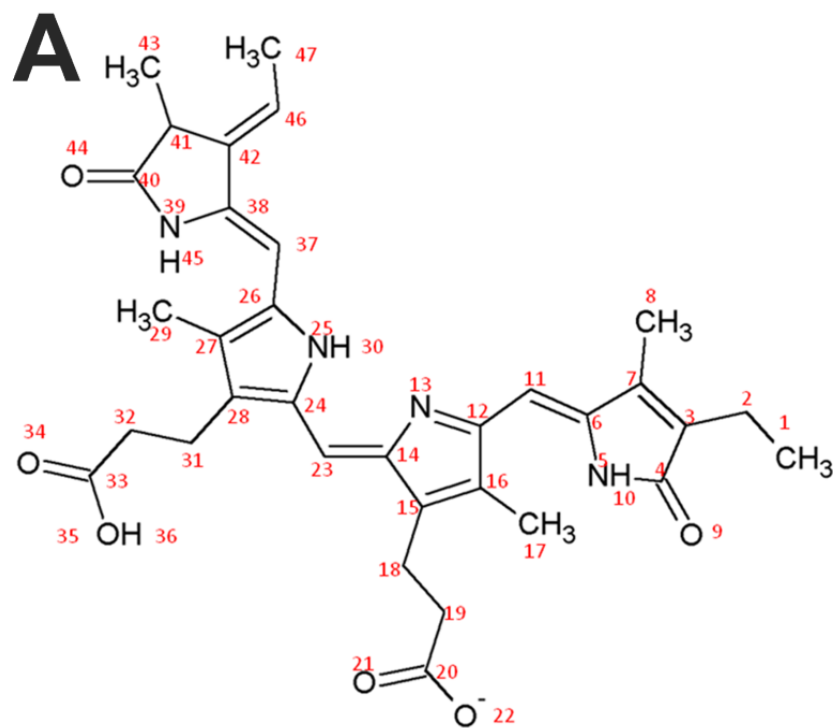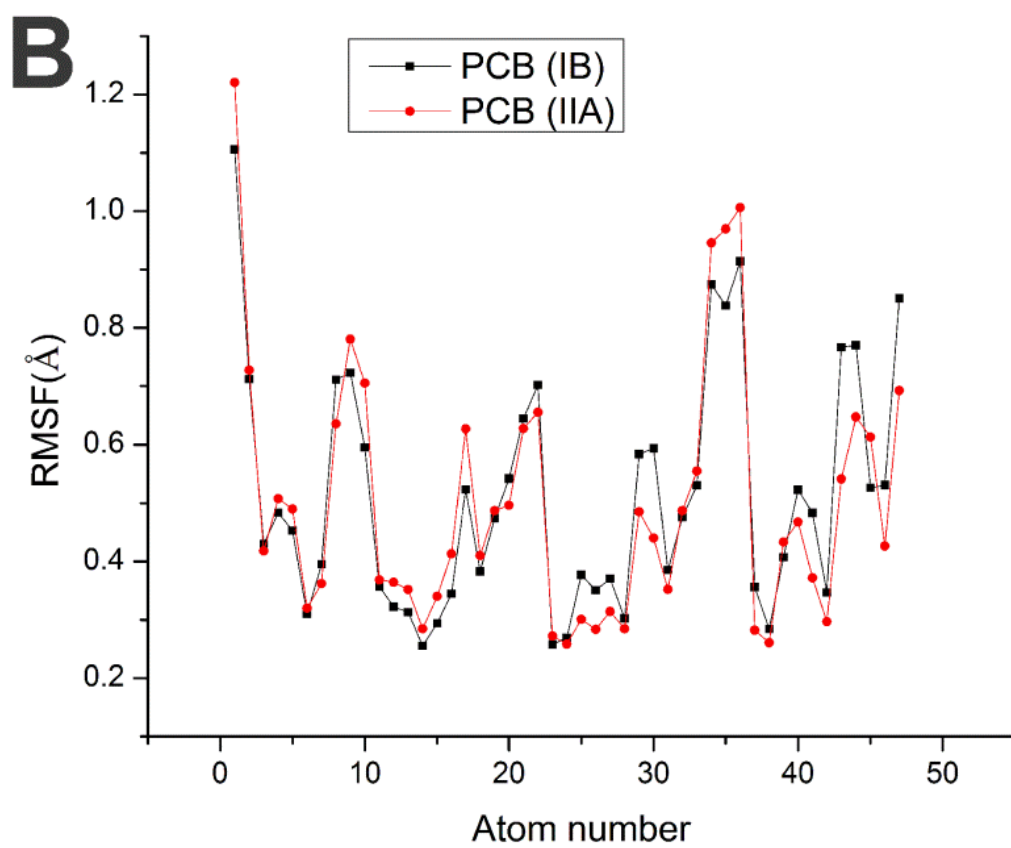

Supplement: S6 Fig — (PDF) [file pone.0167973.s006.pdf]

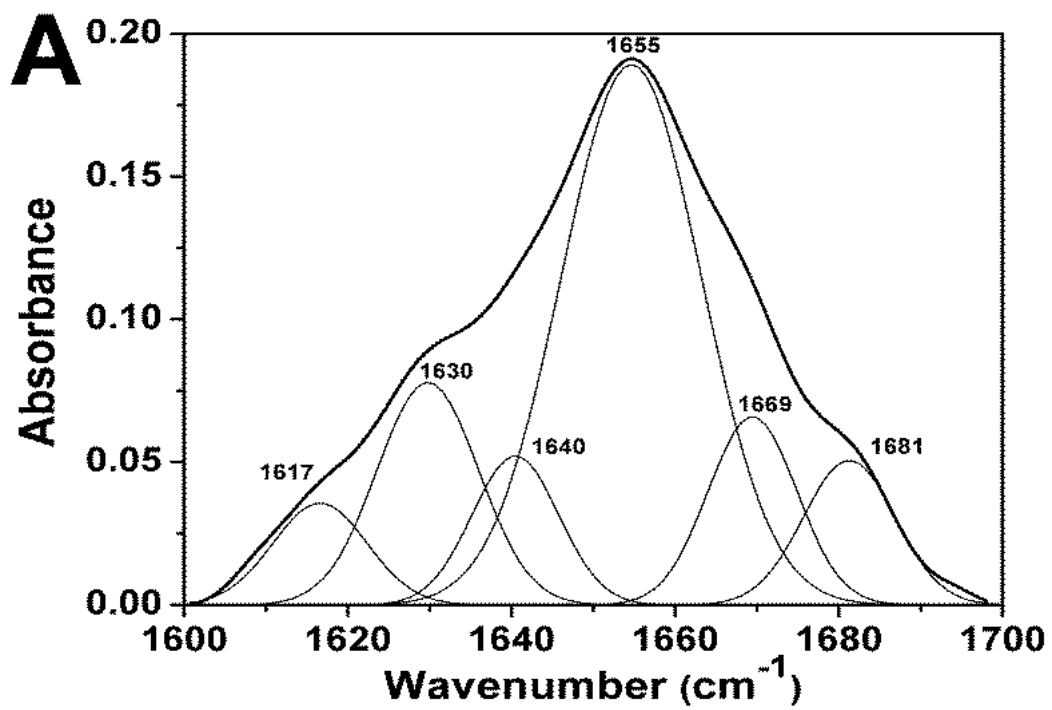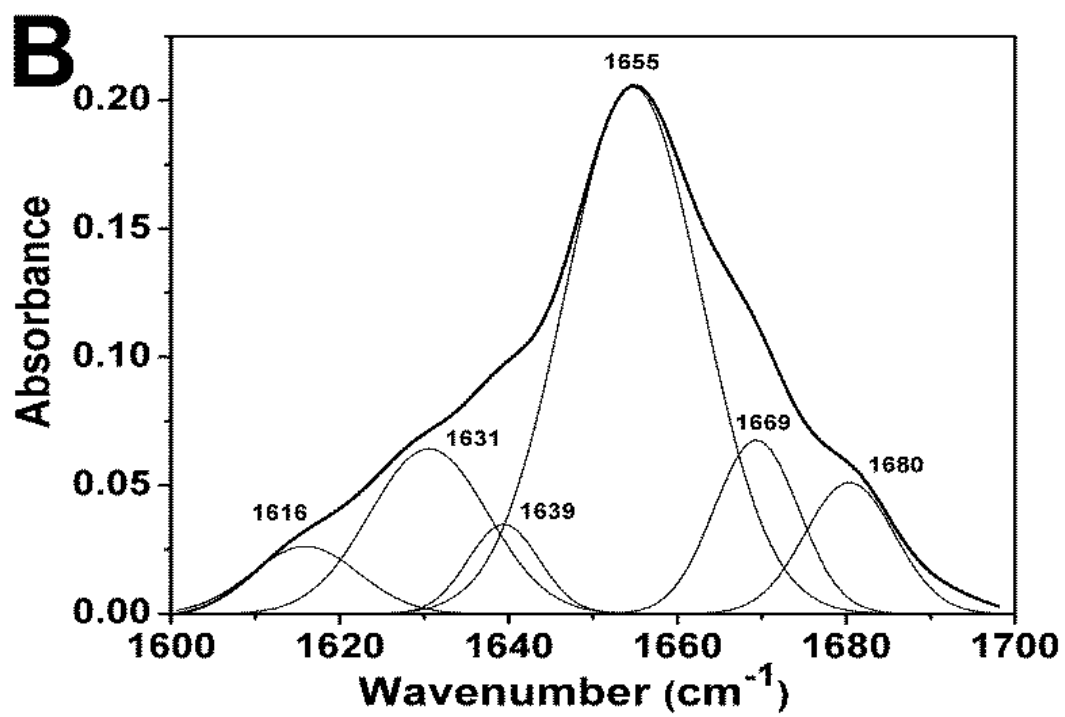

Supplement: S7 Fig — (A) The curve-fit amide I (1700–1600 cm-1) region with secondary structure determination of the free HSA, and (B) the curve-fit amide I (1700–1600 cm-1) region with secondary structure determination of HSA-PCB complex (18 μM both). (PDF) [file pone.0167973.s007.pdf]

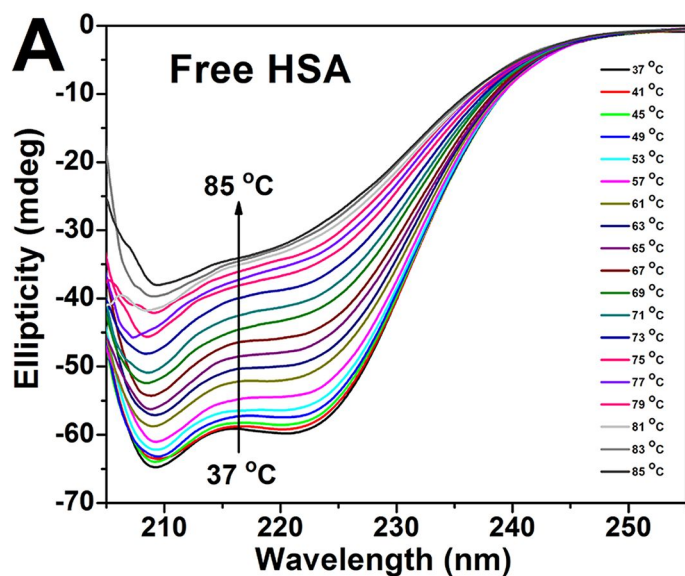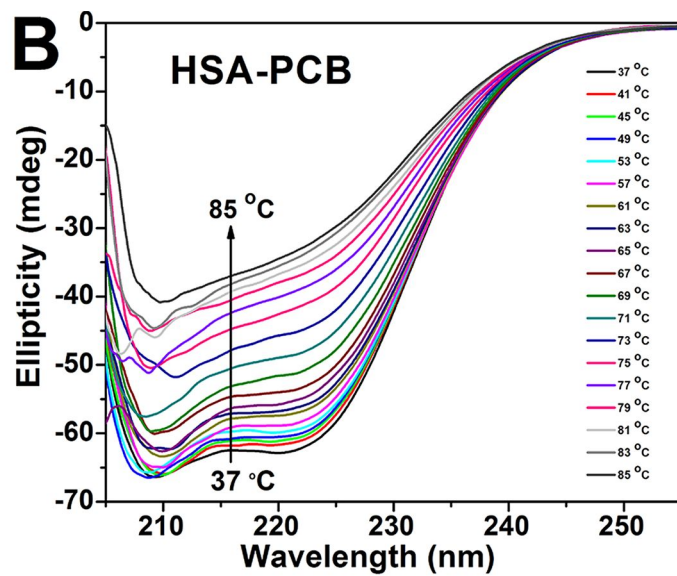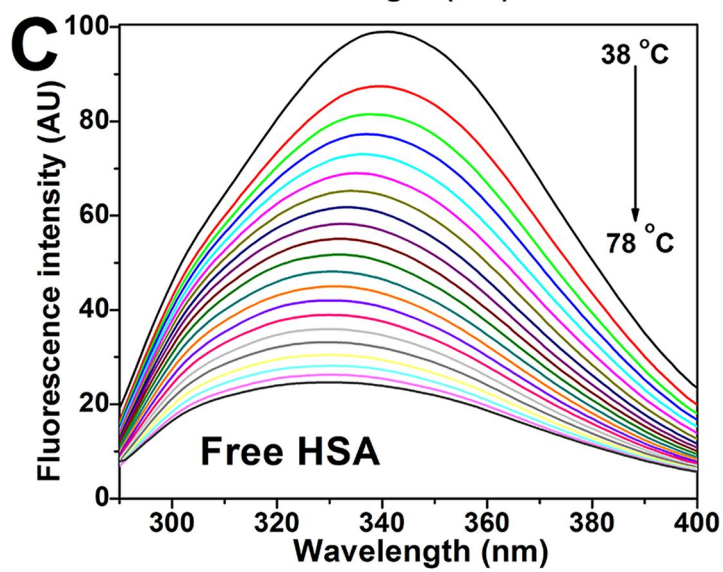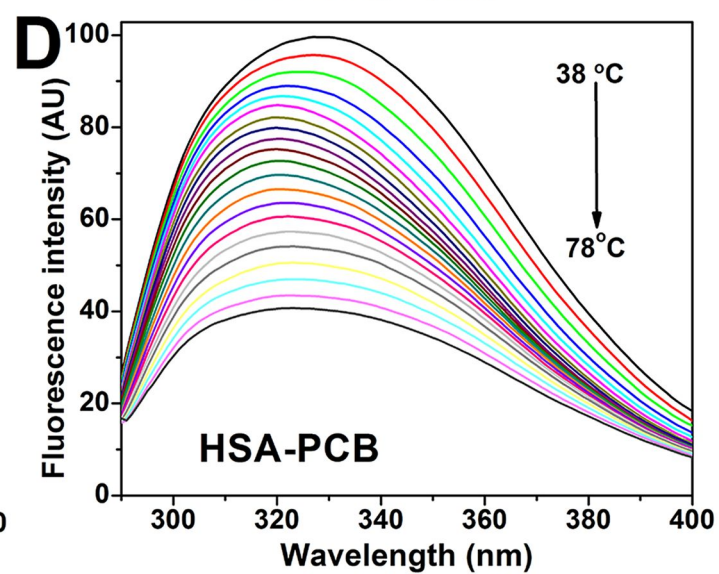

Supplement: S8 Fig — Temperature dependence of 0.5 μM HSA far-UV CD spectra in the presence (A) and absence (B) of 0.5 μM PCB. Note: More pronounced effect of PCB-induced thermal stabilization of HSA, obtained on the basis of an analysis of far-UV CD spectral data, compared to ellipticity at 222 nm at different temperatures is due to differences in time the sample spent at each temperature. Ellipticity at 222 nm was recorded immediately after 1 min of mixture equilibration; CD spectral data were obtained after a total 2 min: 1 min of equilibration, and 1 min of spectra recording. Temperature dependence of 1 μM HSA fluorescence emission spectra in the presence (C) and absence (D) of 1 μM PCB (λ ex = 280 nm). Note: The blue shift of emission maximum in HSA-PCB sample is the result of PCB binding to HSA, as described in our previous study [13]. (PDF) [file pone.0167973.s008.pdf]

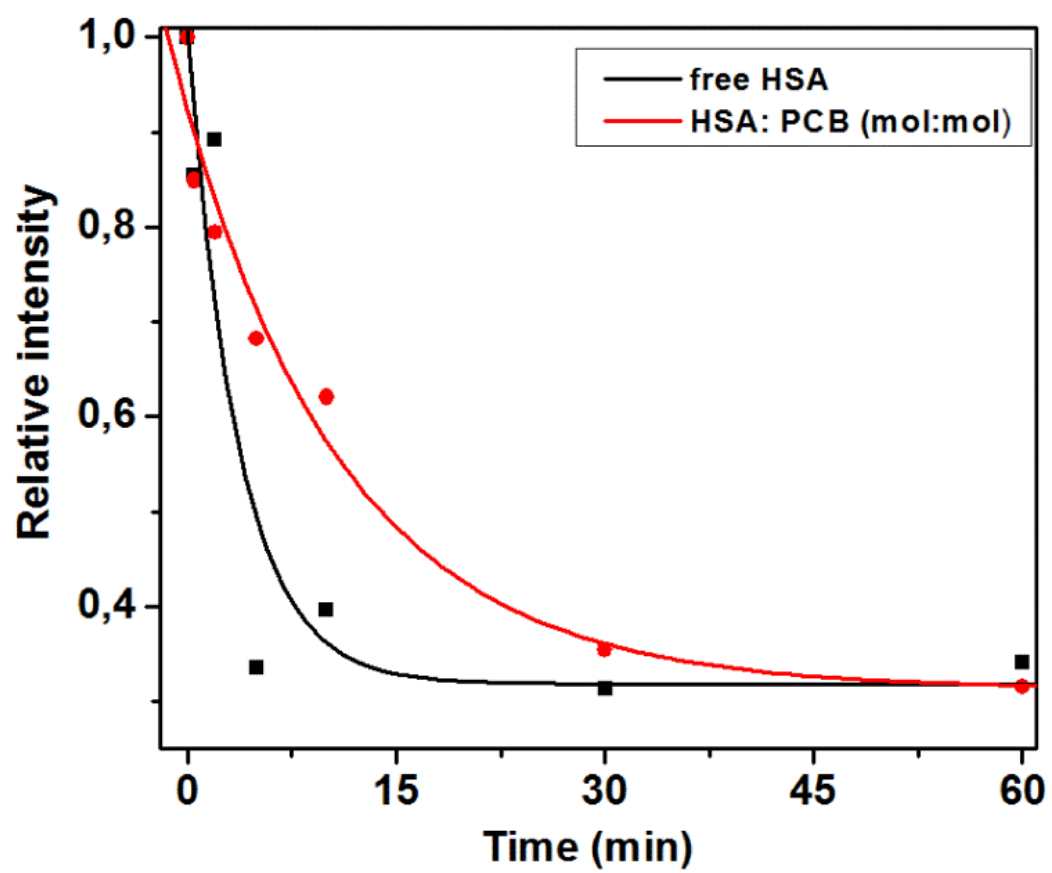

Supplement: S9 Fig — Band intensities at 66 kDa were quantified by densitometry after SDS-PAGE. (PDF) [file pone.0167973.s009.pdf]
