# Supplementary material for: Stabilization of Human Serum Albumin by the Binding of Phycocyanobilin, a Bioactive Chromophore of Blue-Green Alga Spirulina: Molecular Dynamics and Experimental Study
Source: PLoS One. 2016 Dec 13;11(12):e0167973. doi: 10.1371/journal.pone.0167973 (PMC5154526; doi:10.1371/journal.pone.0167973)
Supplement: S1 Table — (PDF) [file pone.0167973.s010.pdf]

Type of interaction	Binding site IIA	Binding site IB
Hydrogen bonds	R222, R257, R218	R117, E141, Y161
Salt bridges	K199	R145, R186
Stacking/CH-π	R218, W214, F211	R186, E141
Hydrophobic	L238, I290, A291	L115, P118, F165, L178, L182
