# Supplementary material for: Stabilization of Human Serum Albumin by the Binding of Phycocyanobilin, a Bioactive Chromophore of Blue-Green Alga Spirulina: Molecular Dynamics and Experimental Study
Source: PLoS One. 2016 Dec 13;11(12):e0167973. doi: 10.1371/journal.pone.0167973 (PMC5154526; doi:10.1371/journal.pone.0167973)
Supplement: S2 Table — (PDF) [file pone.0167973.s011.pdf]

**Type of secondary structure**	**Free HSA**	**HSA: phycocyanobilin (1:1; mol:mol)**
α helix (%)	50.4±2.1	55.2±1.8
β sheet (%)	21.1±1.3	20.1±1.5
random coil (%)	8.8±1.3	4.8±0.7
β turn (%)	11.0±0.5	11.0±0.6
β-antiparallel sheet (%)	8.7±0.8	8.9±0.9
