# Supplementary material for: Stabilization of Human Serum Albumin by the Binding of Phycocyanobilin, a Bioactive Chromophore of Blue-Green Alga Spirulina: Molecular Dynamics and Experimental Study
Source: PLoS One. 2016 Dec 13;11(12):e0167973. doi: 10.1371/journal.pone.0167973 (PMC5154526; doi:10.1371/journal.pone.0167973)
Supplement: S1 Text — (PDF) [file pone.0167973.s012.pdf]

Experimental details

Circular dichroism measurements

Mean residue ellipticity (MRE) was calculated from equation [1]:

$$MRE = \frac{\theta}{10rl[HSA]} \ (\mathbf{1})$$

where θ is ellipticity in mdeg at 209 nm, r is number of amino acid residues (585), l is the path length of the cell in cm (0.01), and [HSA] is molar concentration of protein.

The α-helical content of free and bound HSA (HSA-PCB complex) was calculated from MRE values at 209 nm using the following equation [1]:

$$\%\alpha - helix = \frac{-MRE_{209nm} - 4000}{33000 - 4000} x100 \ (\mathbf{2})$$

FT-IR spectroscopy measurements

Spectra were collected *via* the ATR method with a resolution of 2 cm^{-1} and 64 scans. The infrared spectra of HSA (18 μM) and the HSA-PCB equimolar complex (18 μM both) were obtained in the featured region of 1700-1500 cm^{-1}. Corresponding absorbance contribution of buffer and free ligand solution were recorded and subtracted to get the FT-IR spectra of protein, and of HSA-PCB complex, respectively. The protein secondary structure composition was determined from the shape of the amide I band, located around 1650-1660 cm^{-1}.

Fourier self deconvolution and secondary derivative were applied to the range of 1700-1600 cm^{-1} to estimate the number, position, and areas of component bands. The peaks corresponding to β-sheet (1615-1637 cm^{-1}), random coil (1638-1648 cm^{-1}), α-helix (1649-1660 cm^{-1}), β-turn (1660-1680 cm^{-1}), and β-antiparallel sheet (1680-1692 cm^{-1}) were adjusted and the

area was measured with the Gaussian function [2]. The area of individual band assigned to a given secondary structure was then divided by the total area. The curve fitting process was carried out by PeakFit 4.12 software (SeaSolve software Inc., USA) to get the optimal Gaussian shaped curves that fit the original protein spectrum.

1. Matei I, Hillebrand M. Interaction of kaempferol with human serum albumin: a fluorescence and circular dichroism study. J Pharm Biomed Anal. 2010; 51(3):768−73.

2. Cheng H, Liu H, Bao W, Zou G. Studies on the interaction between docetaxel and human hemoglobin by spectroscopic analysis and molecular docking. J Photochem Photobiol B. 2011; 105:126−132.
