# Supplementary material for: Stabilization of Human Serum Albumin by the Binding of Phycocyanobilin, a Bioactive Chromophore of Blue-Green Alga Spirulina: Molecular Dynamics and Experimental Study
Source: PLoS One. 2016 Dec 13;11(12):e0167973. doi: 10.1371/journal.pone.0167973 (PMC5154526; doi:10.1371/journal.pone.0167973)
Supplement: S2 Text — (PDF) [file pone.0167973.s013.pdf]

Binding sites for PCB on HSA

As we previously stated from our molecular docking studies, PCB could bind at two different binding sites of HSA: IB and IIA [1]. In order to investigate number and type of various interactions involved in stabilization of PCB-HSA complex we thoroughly examined simulated systems. Moreover, molecular dynamics (MD) simulation study enabled us to refine previously proposed binding sites for PCB and better define interactions at the binding site and ligand conformation upon protein binding.

At binding site IIA (Table S1 and Fig S1A) residue K199 forms two salt bridges with carboxyl and carboxylate groups of PCB, with distance of 1.78 Å and 1.64 Å, respectively. Numerous hydrogen bonds additionally contribute to the stability of bound PCB to IIA binding site. There are hydrogen bonds between R222 and deprotonated pyrrole ring, lactam oxygen atom is stabilized by bifurcated interaction of R257 amino groups. Carboxylate group of ligand is stabilized by hydrogen bonding with R218. Residues R218, W214 and F211 are involved in π-interactions and hydrophobic contribution to the stabilization of PCB in IIA binding site arises from residues L238, I290 and A291.

PCB bound at IB binding site (Table S1 and Fig S1B) is stabilized mostly by the various hydrogen bonds. Residue R117 forms bifurcated hydrogen bond with protonated pyrrole ring of PCB. Residues E141 and Y161 interact with lactam ring, whereas E141 is additionally involved in hydrogen bonding with protonated pyrrole ring. Salt bridge is formed between carboxylate group of PCB and amino groups of residues R145 and R186 with the bond length of 1.65 Å and 1.64 Å, respectively. Pyrrole ring is also involved in π-interactions with residues R186 and E141, while hydrophobic interactions involve residues L115, P118, F165, L178 and L182.

1. Minic SL, Milcic M, Stanic-Vucinic D, Radibratovic M, Sotiroudis TG, Nikolic MR, et al. Phycocyanobilin, a bioactive tetrapyrrolic compound of blue-green alga Spirulina, binds with high affinity and competes with bilirubin for binding on human serum albumin. RSC Adv. 2015; 5(76):61787-98.
