# Supplementary material for: Stabilization of Human Serum Albumin by the Binding of Phycocyanobilin, a Bioactive Chromophore of Blue-Green Alga Spirulina: Molecular Dynamics and Experimental Study
Source: PLoS One. 2016 Dec 13;11(12):e0167973. doi: 10.1371/journal.pone.0167973 (PMC5154526; doi:10.1371/journal.pone.0167973)
Supplement: S3 Text — (PDF) [file pone.0167973.s014.pdf]

PCB induces changes in FT-IR spectrum of HSA

Infrared spectra of proteins exhibit a number of the amide bands, representing different vibrations of the peptide moiety. The amide I peak position occurs in the region 1600-1700 cm^{-1} (mainly C=O stretch) and amide II peak in the region 1550-1500 cm^{-1} (C-N stretch coupled with N-H bending mode). Amide I band is more sensitive to the change of protein secondary structure than amide II [1]. Fig 4C (in the main article) shows FT-IR spectra of HSA in the presence and absence of the PCB. There is a negligible spectral peak position shift of amide I band and amide II band, with a change (widening) of the amide I band shape, indicating that the secondary structure of HSA is altered when PCB is added in the equal molar ratio. A quantitative analysis of the HSA secondary structures before and after the interaction with PCB in Tris buffer, pH 7.4 is given in Fig S7 and Table S2. The assignment of the component bands for the protein amide I band were as described in the main text. The percentage of each secondary structure of protein can be calculated depend on the integrated areas of the component bands.

1. Bakkialakshmi S, Barani V. FTIR study on the interaction of quercetin and amantadine with egg albumin. In J Phar Chem Biol Sci. 2013; 3:559−64.
